# Metabolic Flexibility as a Major Predictor of Spatial Distribution in Microbial Communities

**DOI:** 10.1371/journal.pone.0085105

**Published:** 2014-01-21

**Authors:** Franck Carbonero, Brian B. Oakley, Kevin J. Purdy

**Affiliations:** 1 School of Life Sciences, University of Warwick, Coventry, United Kingdom; 2 Department of Food Science, University of Arkansas, Fayetteville, Arkansas, United States of America; 3 United States Department of Agriculture, Agricultural Research Service, Richard B. Russell Research Center, Athens, Georgia, United States of America; Mount Allison University, Canada

## Abstract

A better understand the ecology of microbes and their role in the global ecosystem could be achieved if traditional ecological theories can be applied to microbes. In ecology organisms are defined as specialists or generalists according to the breadth of their niche. Spatial distribution is often used as a proxy measure of niche breadth; generalists have broad niches and a wide spatial distribution and specialists a narrow niche and spatial distribution. Previous studies suggest that microbial distribution patterns are contrary to this idea; a microbial generalist genus (*Desulfobulbus*) has a limited spatial distribution while a specialist genus (*Methanosaeta*) has a cosmopolitan distribution. Therefore, we hypothesise that this counter-intuitive distribution within generalist and specialist microbial genera is a common microbial characteristic. Using molecular fingerprinting the distribution of four microbial genera, two generalists, *Desulfobulbus* and the methanogenic archaea *Methanosarcina*, and two specialists, *Methanosaeta* and the sulfate-reducing bacteria *Desulfobacter* were analysed in sediment samples from along a UK estuary. Detected genotypes of both generalist genera showed a distinct spatial distribution, significantly correlated with geographic distance between sites. Genotypes of both specialist genera showed no significant differential spatial distribution. These data support the hypothesis that the spatial distribution of specialist and generalist microbes does not match that seen with specialist and generalist large organisms. It may be that generalist microbes, while having a wider potential niche, are constrained, possibly by intrageneric competition, to exploit only a small part of that potential niche while specialists, with far fewer constraints to their niche, are more capable of filling their potential niche more effectively, perhaps by avoiding intrageneric competition. We suggest that these counter-intuitive distribution patterns may be a common feature of microbes in general and represent a distinct microbial principle in ecology, which is a real challenge if we are to develop a truly inclusive ecology.

## Introduction

In order to better understand the ecology of microbes and their role in the global ecosystem, it is essential to determine whether ecological ideas and theories that have been derived from studies on plants and animals are also applicable to microbes [1]–[4]. However, it may be that, due to differences in scale and physiologies between micro- and macroorganisms, there will be principles of ecology that are difficult to reconcile between the two. Developing an inclusive ecology represents a substantial challenge as for many years a primary assumption in microbial ecology was that while all microbes could be found in all environments, community structures were shaped by local environmental factors, exemplified in Baas-Becking's statement “*Everything is everywhere, but the environment selects*” [5]. In contrast traditional ecology, where endemism and biogeography are the norm, can be exemplified by the ecological truism that “*all species are always absent from almost everywhere*” [6]. However, microbial communities have been shown to have distinct spatial distribution patterns [7]–[11], yet it is unclear what processes structure microbial communities or whether ecological ideas, such as biogeography [12], [13], are readily applicable to microbes.

The direct application of ecological theory to microbes is difficult because of the complexity of defining microbial taxonomic groups and the extent of microbial genetic and phenotypic diversity [14], [15]. These problems particularly confound the whole bacterial assemblage analyses that are commonly used in microbial ecology. Such broad-brush studies have no equivalent in traditional ecology where studies are usually constrained within tight taxonomic boundaries. Ecologists avoid the problems common to studies in microbial ecology as they use these model species/genera to represent larger groups [16], [17]. Such an approach has rarely been applied to microorganisms primarily because of the difficulty in defining homogeneous species. This issue can be avoided by studying model microbial genera if the selected genera are phylogenetically and functionally homogeneous [18]. Therefore, to study the environmental distribution of microbes we selected model genera that fitted these specific criteria and could also be defined as either specialists or generalists.

A specialist or generalist organism is most succinctly defined as having either a narrow or wide potential niche respectively. However, defining the actual extent of an organism's niche, the n-dimensional space that affects an organism's growth and survival [19], is clearly extremely challenging, if not impossible. Therefore, within traditional ecology spatial distribution has become an accepted proxy measure of niche breadth for many taxa, so it is assumed that a specialist organism with a narrow niche will have a limited spatial distribution and a generalist a wide spatial distribution [20]–[22]. In microbial ecology such assumptions, which underpin many ideas in ecology, have never been properly tested. There are microbes that utilise a vast array of carbon sources for energy and growth and are also capable of respiring more than one type of compound, thus are metabolically flexible with multiple electron donors and acceptors and so can confidently be defined as generalists with wide potential niches. Conversely, there are microbes that have very specific and limited metabolic needs and are clearly highly specialised with narrow potential niches. Thus, metabolic flexibility represents a fundamentally important aspect of a microbe's ecological function and, we propose, represents a reasonable proxy for niche breadth. Therefore, if the spatial distribution of metabolically generalist and specialist microbial model genera is a good proxy for niche breadth this would be a strong indicator that some rules that govern the distribution of large organisms such as plants and animals can also be applied to microbes.

We tested the idea that the extent of spatial distribution along an estuarine gradient is related to metabolic flexibility in two model microbial genera that are both distributed along the full length of the Colne estuary, Essex, UK [23], [24]. Estuaries are natural environmental gradients that have been used to show spatial and evolutionary differentiation in macro-organisms [17], [25] and are hotspots of biogeochemical cycling and microbial activity [26]. The model genera were the sulfate-reducing bacteria *Desulfobulbus*, a generalist genus that can respire both sulfur and nitrogen oxyanions, use fermentation for growth and metabolise a range of carbon sources [27], [28] and the methanogenic archaeal genus *Methanosaeta*, a metabolic specialist that uses acetate as both electron acceptor and donor and as its sole carbon source [29]. Genotypes (at approximately the species level) of the generalist *Desulfobulbus* genus were spatially restricted to distinct regions of the estuarine salinity gradient in a manner similar to that classically described for estuarine macrofauna [17] while genotypes of the specialist *Methanosaeta* showed no such differential distribution and were monotonically distributed along the estuary [8], [30]–[32].

These data challenge assumptions from traditional ecology that spatial distribution is a proxy measure of niche breadth leading us to propose the hypothesis that, in contrast to traditional ecological ideas, the breadth of the spatial distribution of specialist and generalist microbes is inversely related to the breadth of their potential niches. However, this hypothesis is derived from data from only two genera that are from different Domains and have very different metabolisms and so we cannot exclude the possibility that the differences seen in their distributions are due to their phylogenetic or metabolic differences and not differences in their metabolic flexibility. Therefore, to test the hypothesis above and to determine whether metabolic flexibility in general does influence the spatial distribution of microbes we expanded this study to include the most generalist methanogenic archaeal genus, *Methanosarcina*, and the highly specialised sulfate-reducing bacterial genus, *Desulfobacter. Methanosarcina* is the only methanogen genus able to perform all three methanogenic respiratory pathways [33] while *Desulfobacter* are SRB that respire sulfur oxyanions only, primarily whilst oxidising acetate [34]. Analysis of these additional genera produces a dataset that includes a generalist and specialist genus from both the bacterial and the archaeal Domains and that are methanogens and sulphate-reducers and so will address whether the specific metabolism or phylogeny of these four model genera or indeed their metabolic flexibility is a driver of the counter-intuitive differential distribution of *Methanosaeta* and *Desulfobulbus* detected previously.

## Results

**Figure 1:**
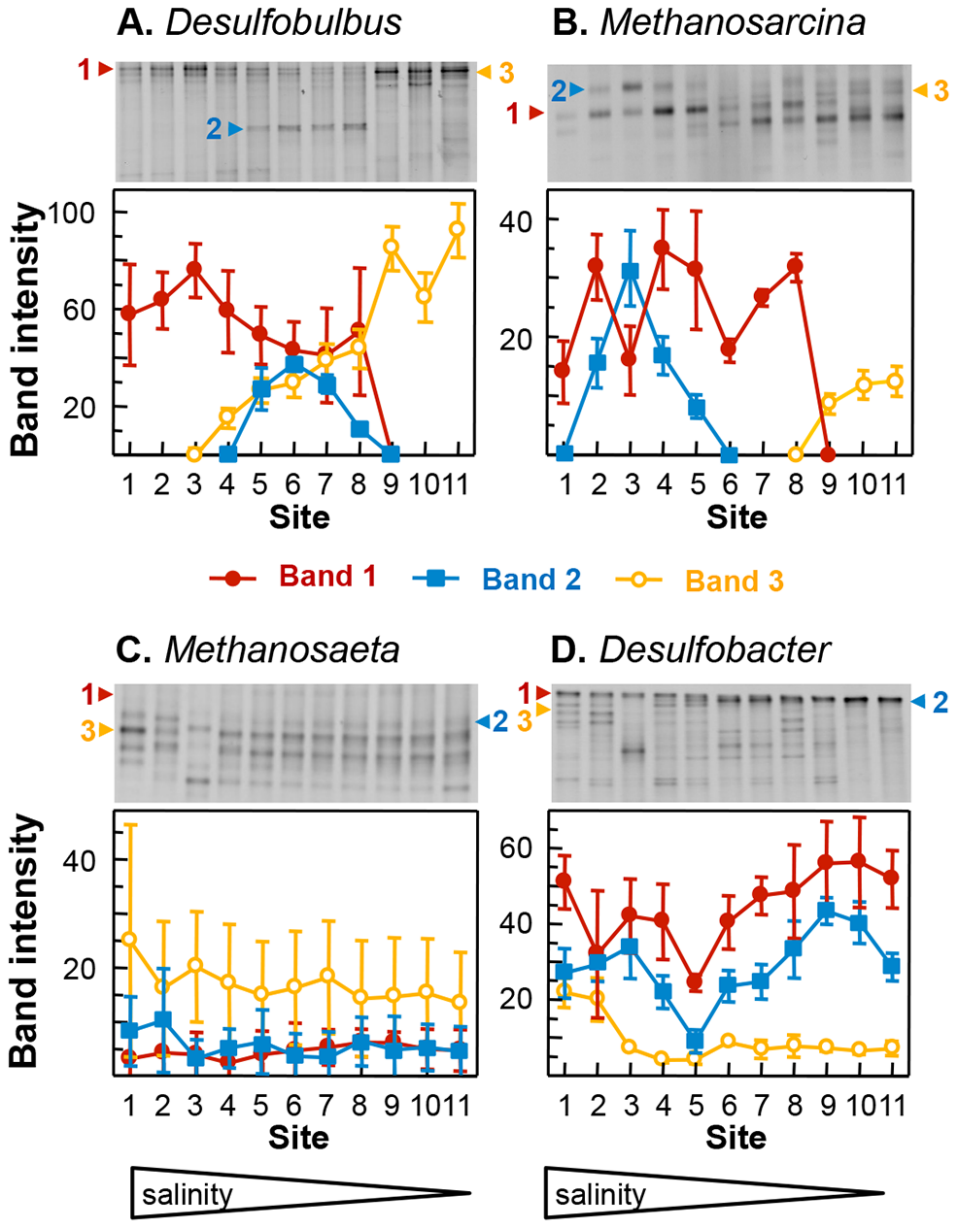
Corrected mean band intensities of representative bands from replicated DNA-DGGE analyses from the 11 sites (from the marine site 1 to the freshwater site 11) along the Colne estuary, Essex, UK for: A. *Desulfobulbus*, B. *Methanosarcina*, C. *Methanosaeta* and D. *Desulfobacter*. Numbered arrows on each gel image indicate band numbers. [Band 1 (line with solid circle) Band 2 (line with open square) Band 3(line with open circle)]. Error bars represent the standard error of the mean (SEM, n = 3). A complete profile is given in Figure S3.

16S rRNA gene fragments, amplified by genus-specific PCR (Table S1 in File S1), were used to analyse the distribution of all four genera in sediment along the full length of the Colne estuary, Essex, UK (Figure S1) using the molecular fingerprinting method Denaturing Gradient Gel Electrophoresis (DGGE; Supplementary information). Profiles of representative bands are shown in Figure 1 (for complete profiles see Figure S3). Genotype distribution patterns from metabolic generalist (*Methanosarcina* and *Desulfobulbus*) and specialist (*Methanosaeta* and *Desulfobacter*) model genera were highly dissimilar. Both generalist models showed a restricted spatial distribution, in which particular genotypes were only found in certain regions of the estuary, while both specialist models showed no such restricted distribution, with all bands detected all along the estuary.

**Figure 2:**
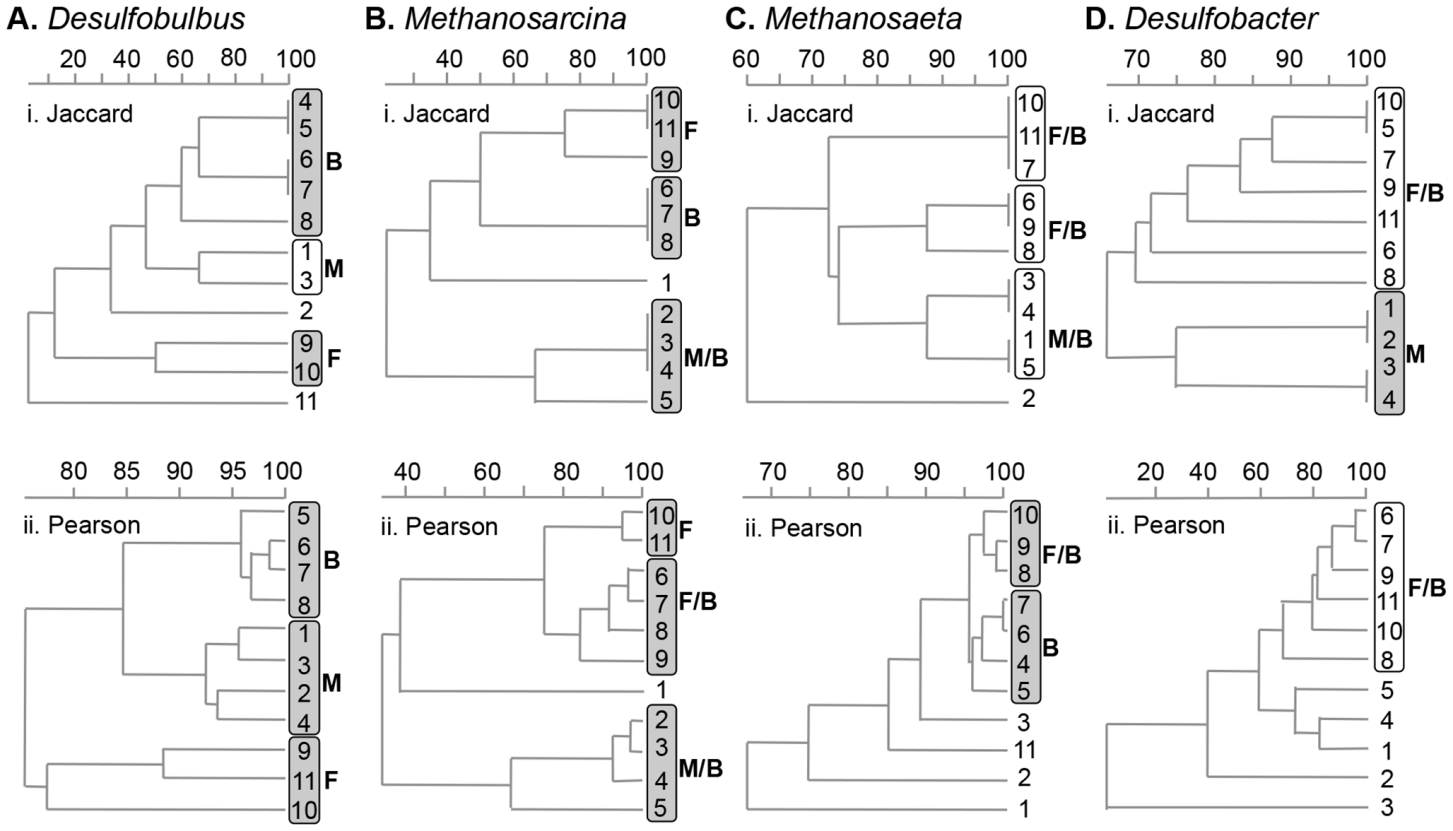
Cluster analyses of the DNA-DGGE profiles: i. Jaccard and ii. Pearson analyses of A. *Desulfobulbus*; B. *Methanosarcina*; C. *Methanosaeta*; D. *Desulfobacter*. Marine (M), Marine/Brackish (M/B), Brackish (B), Freshwater/Brackish (F/B) and Freshwater (F) clusters are circled. Shaded clusters are geographically continuous, unshaded clusters are not.

Cluster analysis of both DGGE band presence/absence (Jaccard) and total DGGE profile (Pearson) indicated geographically coherent clustering for the generalist models, *Desulfobulbus* and *Methanosarcina* (Figure 2A and B). The specialist models, *Methanosaeta* and *Desulfobacter*, showed inconsistent and geographically incoherent clustering with generally high overall similarities between sites (>59%) across the two methods (Figure 2C and D). Unconstrained Canonical Correspondence Analysis (CCA) of the DGGE fingerprints showed some clustering according to site for all genera (Figure S4). However, very low eigenvalues (scales on the CCA; 0.015–0.048) in the CCA of the specialist genera (Figure S4C and D) indicated a poor correlation between genotypic dissimilarity matrixes and environmental variables while eigenvalues (0.181–0.680) were higher for the generalist models (Figure S4A and B). Mantel and partial Mantel tests revealed a significant correlation (*p*<0.05) between the geographic distance between sites and distribution of the generalist models (Table S2A and B in File S1) whether based on Band Intensity, Jaccard or Pearson analyses. Very limited significant correlations were found for *Desulfobacter* and *Methanosaeta* (Table S2C and D in File S1), with no clear coherent correlations across all three methods of analysis.

**Figure 3:**
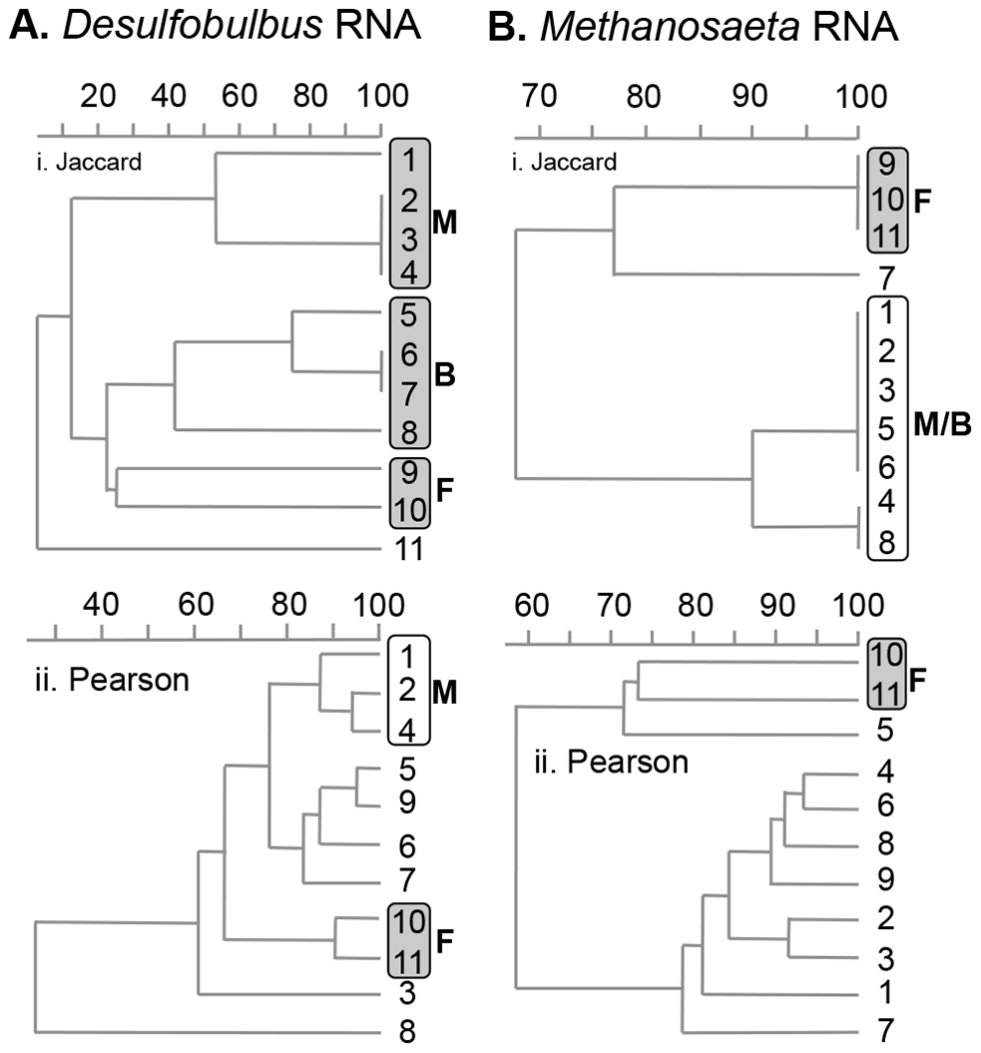
Cluster analyses of the RNA-DGGE profiles: i. Jaccard and ii. Pearson analyses of A. *Desulfobulbus*; B. *Methanosarcina*. Marine (M), Marine/Brackish (M/B), Brackish (B), Freshwater/Brackish (F/B) and Freshwater (F) clusters are circled. Shaded clusters are geographically continuous, unshaded clusters are not.

Analysis of RNA (in this case 16S rRNA) provides an indication that the organisms detected are not only present but also active in an environment. Banding patterns in the RNA-DGGE analysis of both *Desulfobulbus* and *Methanosaeta* (Figure S5) were very similar to those seen in the DNA-DGGE (Figure 1 and Figure S3). As with DNA-DGGE *Desulfobulbus* had a restricted pattern of spatial distribution, while *Methanosaeta* genotypes were constantly detected all along the estuary [30]. Cluster analyses showed spatially coherent clustering for *Desulfobulbus* clusters (Fig 3A) and a weak but inconsistent clustering for *Methanosaeta* (Fig 3B). Unconstrained CCAs showed strong geographic clustering for *Desulfobulbus* (Eigen values 0.381–0.628; Figure S6A) and no clustering for *Methanosaeta* (Eigen values 0.075–0.138; Figure S6B). Mantel and partial Mantel tests (Table S3 in File S1) revealed a clearly significant correlation between geographic distance and the *Desulfobulbus* distribution pattern (*p*<0.05). While all Mantel tests were significant for *Methanosaeta* (*p*<0.05) no coherent correlation could be seen with partial Mantel tests. Importantly, DNA- and RNA-based *Desulfobulbus* distribution patterns were very similar, as observed previously for *Methanosaeta* [30]. This implies that detected genotypes (from analysis of DNA) are active (from analysis of RNA), and thus DNA based DGGE fingerprints represent a satisfactory representation of metabolically active populations. Pyrosequence analysis of functional genes from *Desulfobulbus* (*dsr*A) and *Methanosaeta* (*mcr*A) along the Colne estuary also shows a clear difference in distribution patterns between these two model genera, supporting the conclusions above [32].

These data show that metabolic flexibility does appear to directly affect the distribution of microbial genera; metabolic generalists (*Desulfobulbus* and *Methanosarcina*) have a specialist spatial distribution strongly correlated with environmental variables and metabolic specialists (*Methanosaeta* and *Desulfobacter*) have a generalist spatial distribution along an estuarine gradient that is not correlated to environmental factors.

## Discussion

Here we show that within microbial genera metabolic flexibility appears to have a profound effect on the spatial distribution patterns of those genera, with members of metabolic generalist genera showing a narrower spatial distribution than metabolic specialist genera. These seemingly counter-intuitive distribution patterns suggest that microbes are distributed in ways that differ from most plants and animals and that these differences would have to be accounted for in a truly inclusive ecology. The comparison described here, using two specialist and two generalist genera from across both archaeal and bacterial domains and both the terminal oxidation processes of sulphate reduction and methanogenesis, suggests that neither phylogeny nor metabolism are major driving forces in the detected distribution of these four genera.

The distribution of the generalist methanogen genus *Methanosarcina* was assessed for the first time, to our knowledge, indicating a differential distribution pattern with genotypes restricted to marine, marine-brackish, brackish-freshwater and freshwater regions of the estuary. *Methanosarcina* strains have been isolated from various freshwater and marine environments and isolates are able to use at least two of the three methanogenic pathways (acetoclastic, hydrogenotrophic and methylotrophic methanogenesis), but with important differences in their substrate preference [33]. In an estuary, acetate concentrations are higher in freshwater regions [35] and decrease substantially due to consumption by SRB where sulfate is freely available, which is generally in all but the most freshwater sediments [36]. *Methanosarcina* are known to use one-carbon compounds, such as methylamines, that are more abundant in marine environments [37]. Thus, both the salinity gradient and the variable availability of different substrates (such as acetate and methylamines) along the estuarine gradient could be strong drivers of *Methanosarcina*'s differential spatial distribution, in a similar way to the distribution of *Desulfobulbus* along the estuary [8], [31]. Thus, the distribution patterns of the generalist model genera are similar to those observed for estuarine macroorganisms, such as *Gammarus* [17], [25] and comparable to classic spatial distributions seen in other systems [16].

*Desulfobacter* distribution was also assessed for the first time, to our knowledge. Some bands appear to fluctuate along the estuary, but no correlation was found with the environmental variables, although some weak site-related clustering was observed (Figure 2D). Thus, the only supportable conclusion is that all genotypes were present all along the estuary. Such an undifferentiated distribution pattern, also seen with *Methanosaeta*, may be a general property of metabolically specialist microbes. Thus, these data support the proposed hypothesis that, in contrast to traditional ecological ideas, the breadth of the spatial distribution of specialist and generalist microbes is inversely related to the breadth of their potential niche and indicate that the pattern of spatial distribution of a microbe is directly linked to its metabolic flexibility.

Whilst definitions of specialisation are varied and often context dependent [38] most ecologists define taxa according to the breadth of their niche; specialists have narrow niches and generalists have broad niches with spatial distribution often used as a proxy measure of niche breadth (e.g. [20], [21], [39]). Thus, in many cases organisms are defined as specialists or generalists based on their existing population structure but not directly on their intrinsic biological properties. Such a distribution-based definition of specialist/generalist has never been used to describe microbial populations and in this study the metabolic specialists would be classified as ecological generalists and vice versa. From an ecological perspective it is counter-intuitive that metabolic versatility should limit the spatial distribution of a species and metabolic restriction result in a broader spatial distribution [39] yet, for these four model genera in this estuary, such contrasting patterns of spatial distribution are unequivocal.

Regardless of functional group or phylogeny specific genotypes of generalists are spatially restricted to specific regions of the estuary, whereas specialists are more evenly distributed and seem generally unaffected by gross environmental variables. These data suggest that spatial differentiation in microbes occurs if individuals within a genus harbour sufficient metabolic flexibility to exploit differing conditions along an environmental gradient. Specialists, that by definition would lack the capacity to exploit differing conditions, should exhibit either a highly restricted (distribution is limited by changing environmental factors that preclude growth/survival) or a cosmopolitan distribution along a gradient (environmental factors in the gradient have little or no effect on the distribution of the specialist genera). The later seems to be the case for the specialist genera we have analysed here. This apparent paradox could be explained by the difference in terms of potential and realised niches [21], [38], [39]. We suggest that metabolic specialists are constrained by relatively few factors, usually the availability of one or two specific substrates (in this study, acetate for *Methanosaeta* and acetate and sulfate for *Desulfobacter*). As a consequence while metabolic specialists have a narrow potential metabolic niche they are able to occupy much of this niche space. If, as suggested, microbial specialists already occupy a great extent of their potential niche, there is little niche space that could be occupied by diverging strains. With little ecological space available for speciation intrageneric competition may be highly constrained and so species coexist along environmental gradients. For generalists the opposite is true, genotypes are spatially constrained within a narrow realised niche but have a much wider potential niche (see isolate data in Oakley et al. [32]) and so do have niche space that can be exploited by diverging strains. Therefore, we propose that all generalist microbial genotypes could potentially occupy additional niche space but are constrained from doing so by intense intrageneric competition. Such competition would be enhanced if environmental heterogeneity, such as temporal variation in the availability of electron donors and acceptors, was great, as appears to be the case on a on a microscopic level in many environments (e.g. [40]). Therefore, intrageneric competition, in concert with environmental heterogeneity, may effectively produce barriers to dispersal that facilitate localised adaptation and differentiation between strains. Thus, a generalist phenotype results in very different ecological outcomes in microbial communities compared to those seen in traditional ecological studies of plants and animals.

We further suggest that among the four ecological processes proposed by Hanson *et al.* [41] as drivers of microbial biogeography, speciation through selection by environmental variables is more relevant to generalists. In contrast, it appears that specialists are probably mainly affected by the three other processes: dispersal, mutation and drift; resulting in presumably subtle and small-scale biogeographic patterns. This challenges the primary assumption that mainly abiotic environmental factors must be accounted for to understand the ecology of microbes as there appear to be different ecological rules for different groups.

Most efforts with the aim of including microbes into our existing ecological framework has been based on using broad-brush approached to determine whether microbial communities are similar to plants and animals in their relationships and interactions with the environment. Here we show, using taxonomically focused analyses, that, in contrast to the perceived view in ecology that specialisation can be defined by spatial restriction [22], microbial metabolic specialists have a wider distribution than metabolic generalists. These results illustrate the real challenge we face in integrating microbes within ecological theory and analysis in order to build a truly unified ecology.

## Materials and Methods

### Ethics statement

No permissions were required to sample the estuarine sites used here as they are freely accessible to the public and not on private land.

### Site description and sampling

The sample sites (see Figure S1) and sampling strategy, sediment porewater salinity, Sulfate and acetate measurements (Figure S2) and sample treatment prior to DNA and RNA extraction are described in File S1.

Nucleic acids extraction, 16S rRNA gene PCR and fingerprint analysis of the model genera

DNA and RNA were extracted from all sediment samples using the hydroxyapatite spin-column method [42], [43] and 16S rRNA gene sequences amplified as described in File S1. A new set of primers specific for *Methanosarcina* 16S rRNA gene (Msc214f and Msc613r; Table S1 in File S1) was designed using the web-based primer design software Primer3 (http://frodo.wi.mit.edu/primer3/) and assessed for specificity and sensitivity with ThermoPhyl ([44]; http://go.warwick.ac.uk/thermophyl). Specificity was checked empirically by amplification of *Methanosarcina mazei* (DSM 2053) and *Methanosarcina acetivorans* (DSM 2834) pure culture DNA and using several other archaeal and bacterial negative controls.

Amplified 16S rRNA-gene fragments from both DNA and RNA were analysed using DGGE fingerprinting as described in Information SI. DGGE patterns corresponded extremely well with qPCR and pyrosequence analysis of genotype distribution [32], [45]. In addition RNA-DGGE analysis required only a 2-step PCR but produced almost identical distribution patterns to those from the 3- or 4-step PCRs used in DNA-DGGE analyses, indicating that the additional steps do not introduce artefacts into the analysis. Dissimilarity matrixes were obtained from Pearson correlation and Jaccard analyses of the DGGE gels in GelComparII (Applied Maths, Belgium). Environmental variables were also converted into dissimilarity matrixes and correlations between the two matrices were analysed using Mantel and partial Mantel tests [46]. Canonical Correspondence Analysis (CCA) was used to assess the effect of the environmental gradient on the distribution of organisms as described in File S1.

## Supporting Information

Figure S1 **Map of the Colne estuary, Essex, UK showing the 11 sampling sites along the full extent of the estuary.**
(TIF)

Figure S2 **Porewater concentrations (mM) of A. chloride (salinity); B. sulphate from the 11 sampled sites along the Colne estuary.** Error bars represent the standard error of the mean (SEM, n = 3).
(TIF)

Figure S3 **Corrected mean band intensities from replicated DNA-DGGE analyses from the 11 sites (from the marine site 1 to the freshwater site 11) along the Colne estuary, Essex, UK for: A.** ***Desulfobulbus*** **, B.** ***Methanosarcina*** **, C.** ***Methanosaeta*** **and D.** ***Desulfobacter*** **.** Band numbers are indicated on the left of gel images. Error bars represent the standard error of the mean (SEM, n = 3).
(TIF)

Figure S4 **Unconstrained Canonical Correspondence Analysis tests correlating environmental variables (geographic distance (GD), chloride (Cl) and sulphate (S)) with model genera's genotypic distribution patterns from DNA-DGGE for A.** ***Desulfobulbus*** **, B.** ***Methanosarcina*** **, C.** ***Methanosaeta*** **and D.** ***Desulfobacter*** **. Geographical groupings of samples are circled and labelled.**
(TIF)

Figure S5 **Corrected mean band intensities from replicated RNA-DGGE analyses from the 11 sites (from the marine site 1 to the freshwater site 11) along the Colne estuary, Essex, UK for: A.** ***Desulfobulbus*** **, B.** ***Methanosaeta*** **.** Band numbers are indicated on the left of gel images. Error bars represent the standard error of the mean (SEM, n = 3).
(TIF)

Figure S6 **Unconstrained Canonical Correspondence Analysis tests correlating environmental variables (geographic distance (GD), chloride (Cl) and sulphate (S)) with model genera's genotypic distribution patterns from RNA-DGGE for A.** ***Desulfobulbus*** **, B.** ***Methanosaeta*** **.** Geographical groupings of samples are circled and labelled.
(TIF)

File S1 **Contains Table S1–S3 and detailed description of Materials and Methods used and basic results with associated references.** **Table S1: List of PCR primers and annealing temperatures used in this study.** ^*^TD - These PCRs were Touchdown PCRs. Initial annealing temperature is +10°C and reduces by 1°C per cycle for the first 10 cycles of the PCR. **Table S2. Mantel tests correlating environmental variables (geographic distance (D), chloride (C) and sulphate (S)) with model genera's DNA-DGGE based genotypic distribution patterns (based on Band intensities, Pearson and Jaccard analyses).** **Table S3. Mantel tests correlating environmental variables (geographic distance (GD), chloride (C) and sulphate (S)) with model genera's RNA-DGGE based genotypic distribution patterns (based on Band intensities, Pearson and Jaccard analyses).**
(DOCX)
