# Supplementary figures and images for: Metabolic Flexibility as a Major Predictor of Spatial Distribution in Microbial Communities
Source: PLoS One. 2014 Jan 21;9(1):e85105. doi: 10.1371/journal.pone.0085105 (PMC3897421; doi:10.1371/journal.pone.0085105)

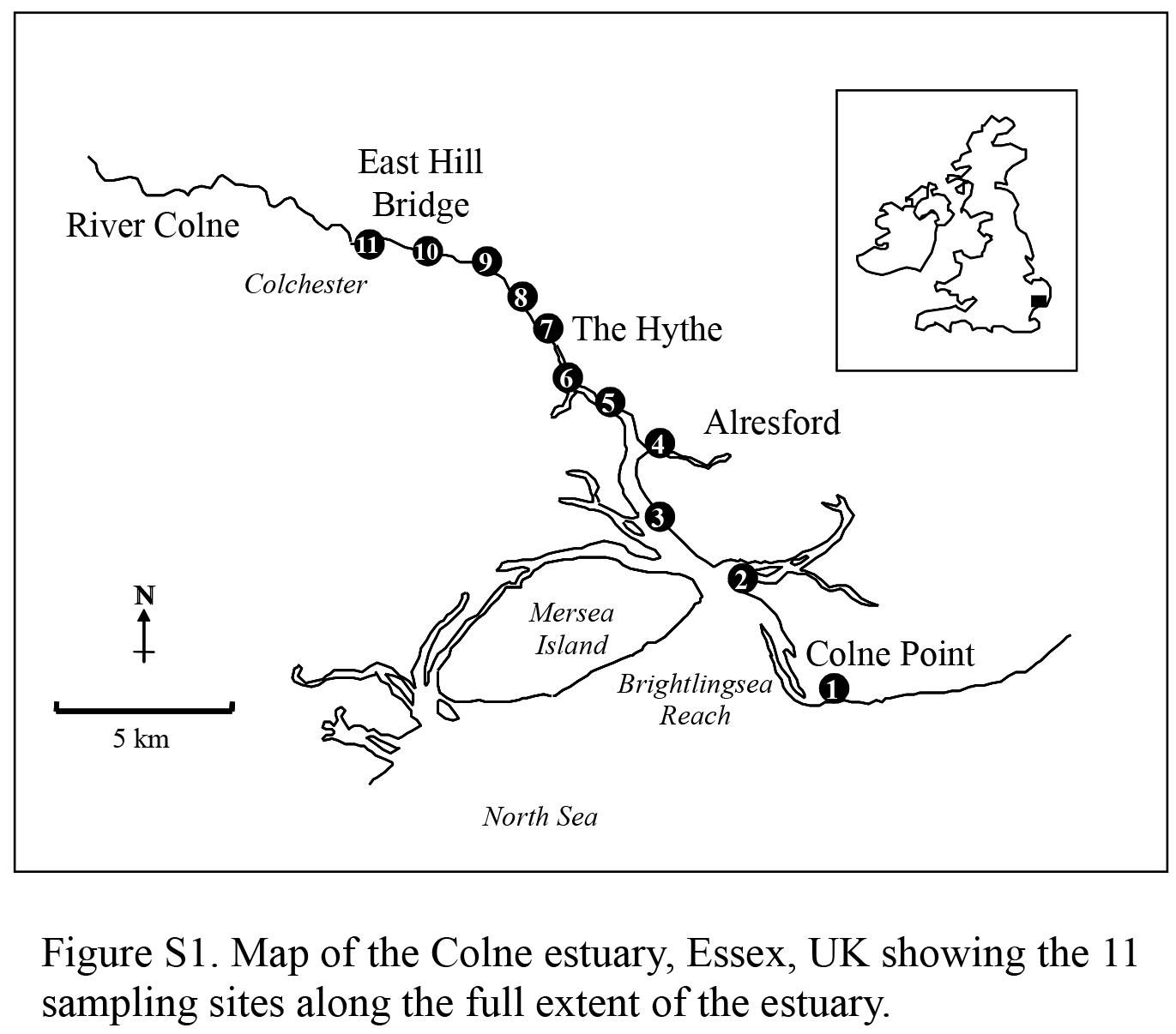

Supplement: Figure S1 — Map of the Colne estuary, Essex, UK showing the 11 sampling sites along the full extent of the estuary. (TIF) [file pone.0085105.s001.tif]

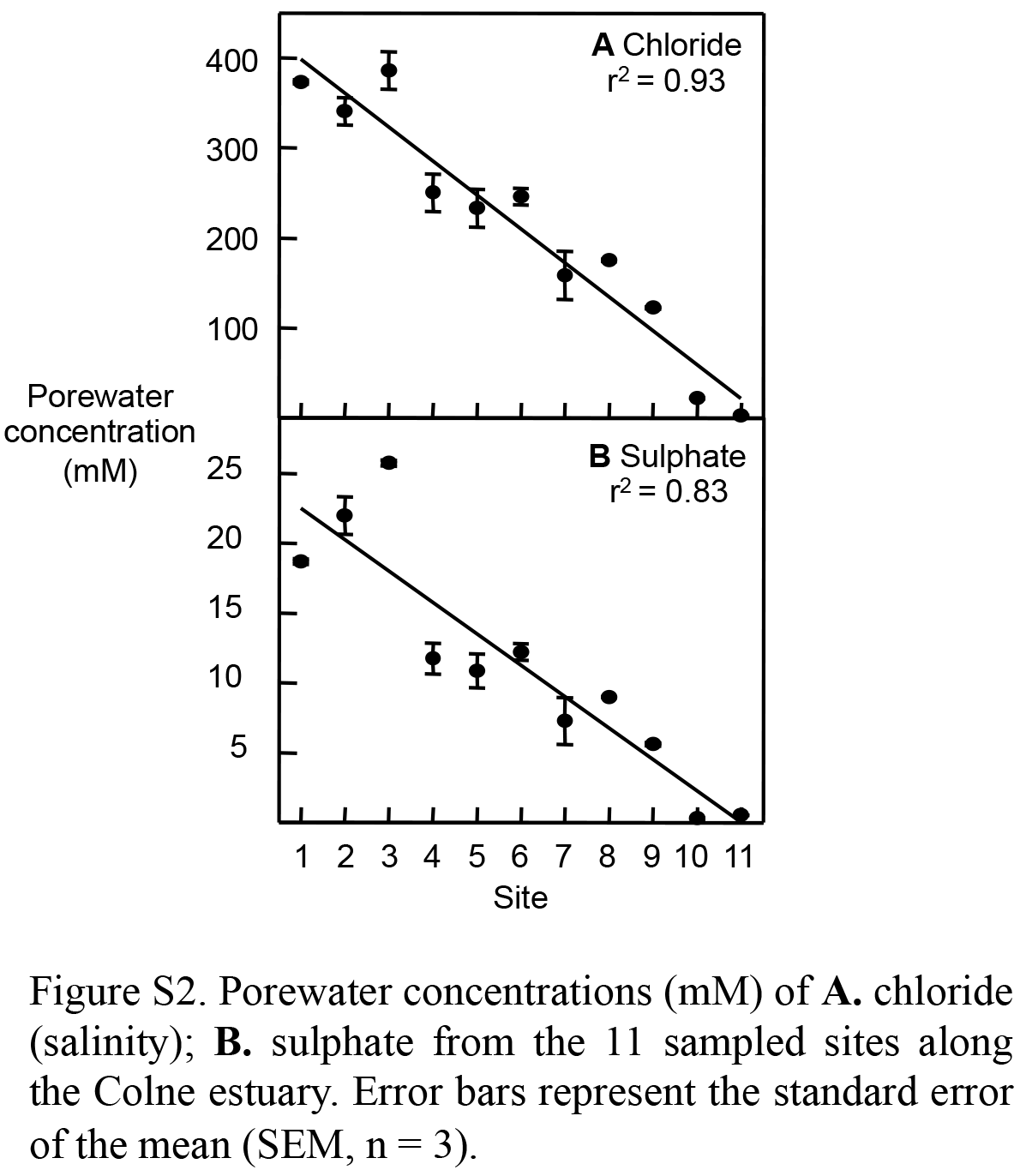

Supplement: Figure S2 — Porewater concentrations (mM) of A. chloride (salinity); B. sulphate from the 11 sampled sites along the Colne estuary. Error bars represent the standard error of the mean (SEM, n = 3). (TIF) [file pone.0085105.s002.tif]

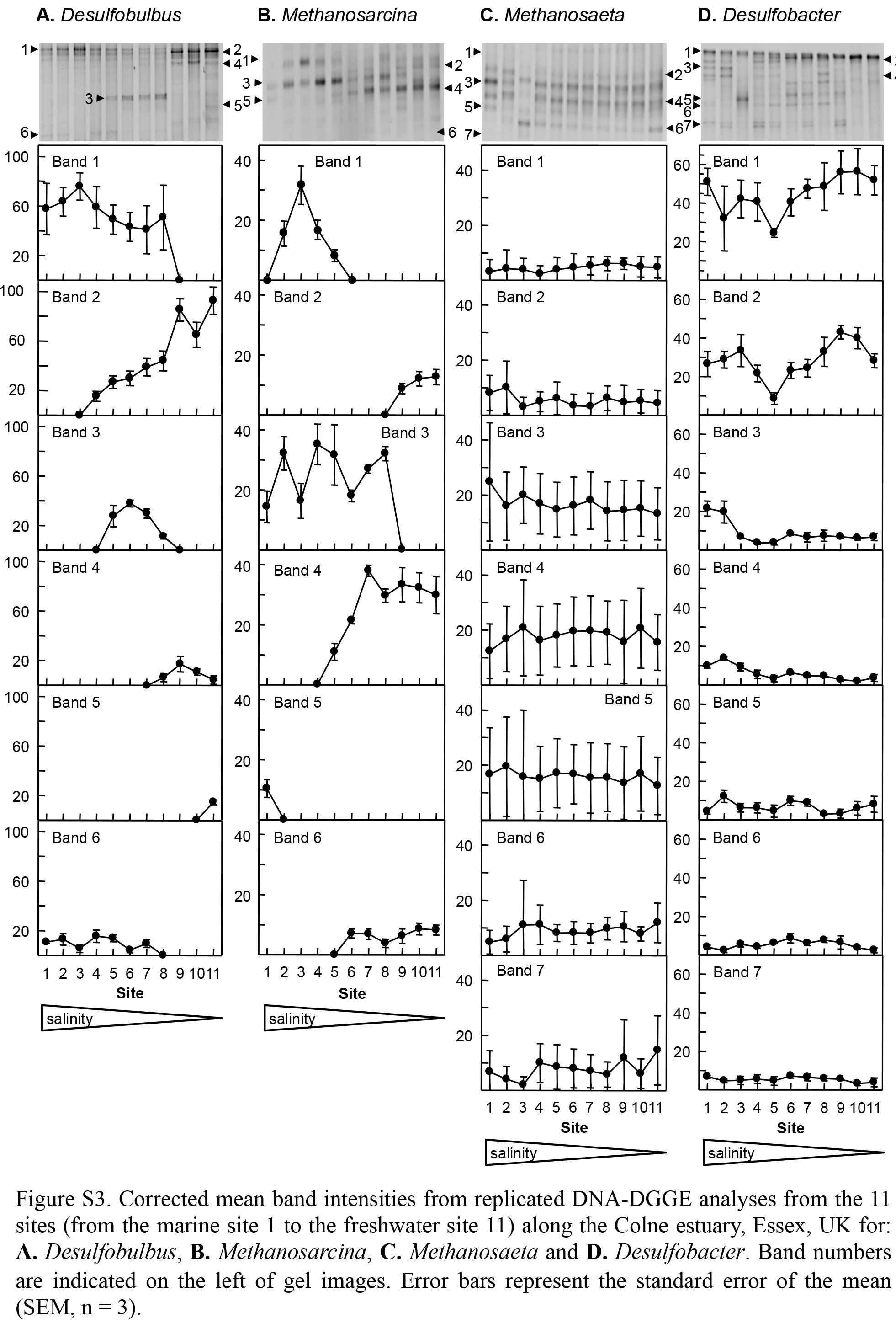

Supplement: Figure S3 — Corrected mean band intensities from replicated DNA-DGGE analyses from the 11 sites (from the marine site 1 to the freshwater site 11) along the Colne estuary, Essex, UK for: A. Desulfobulbus , B. Methanosarcina , C. Methanosaeta and D. Desulfobacter . Band numbers are indicated on the left of gel images. Error bars represent the standard error of the mean (SEM, n = 3). (TIF) [file pone.0085105.s003.tif]

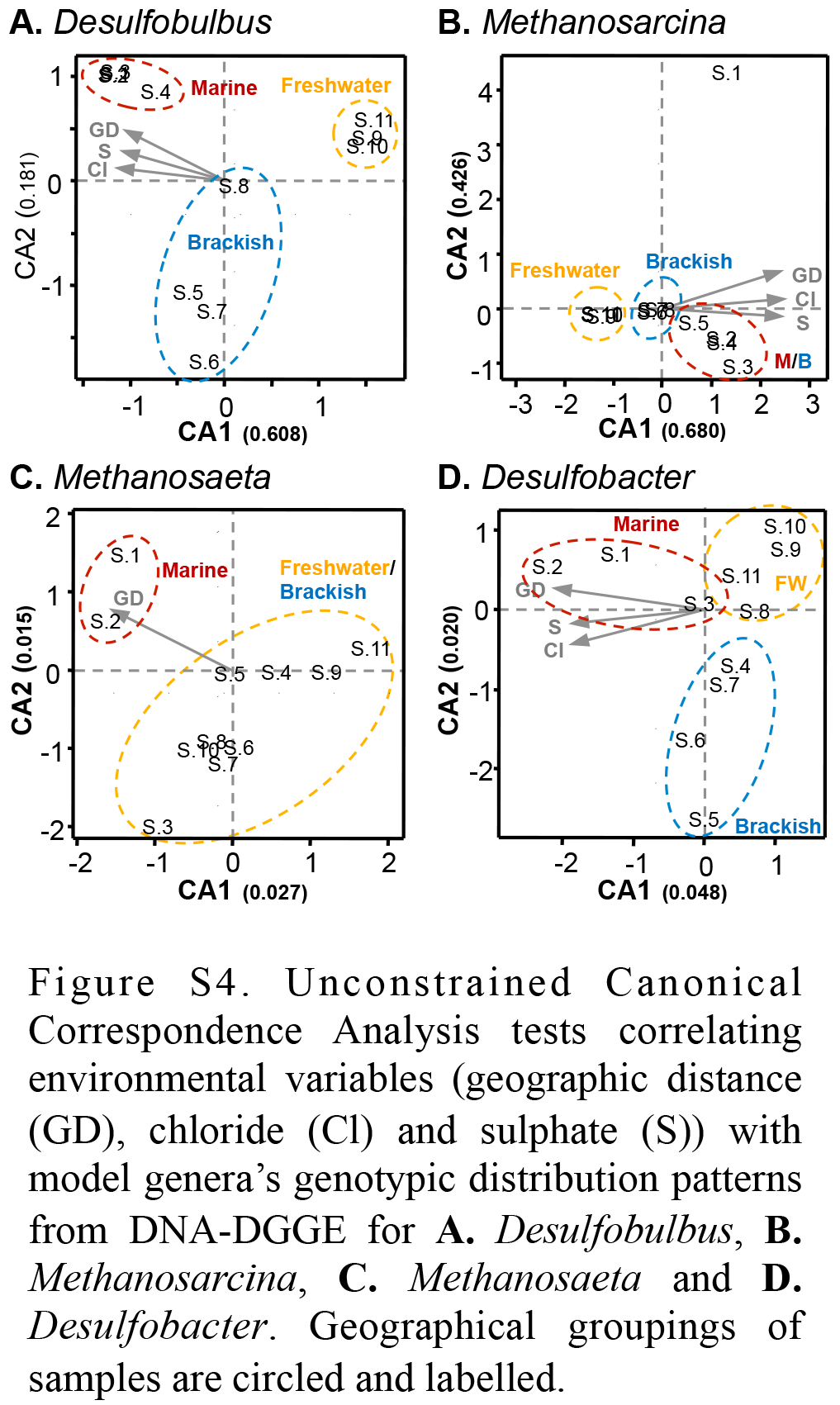

Supplement: Figure S4 — Unconstrained Canonical Correspondence Analysis tests correlating environmental variables (geographic distance (GD), chloride (Cl) and sulphate (S)) with model genera's genotypic distribution patterns from DNA-DGGE for A. Desulfobulbus , B. Methanosarcina , C. Methanosaeta and D. Desulfobacter . Geographical groupings of samples are circled and labelled. (TIF) [file pone.0085105.s004.tif]

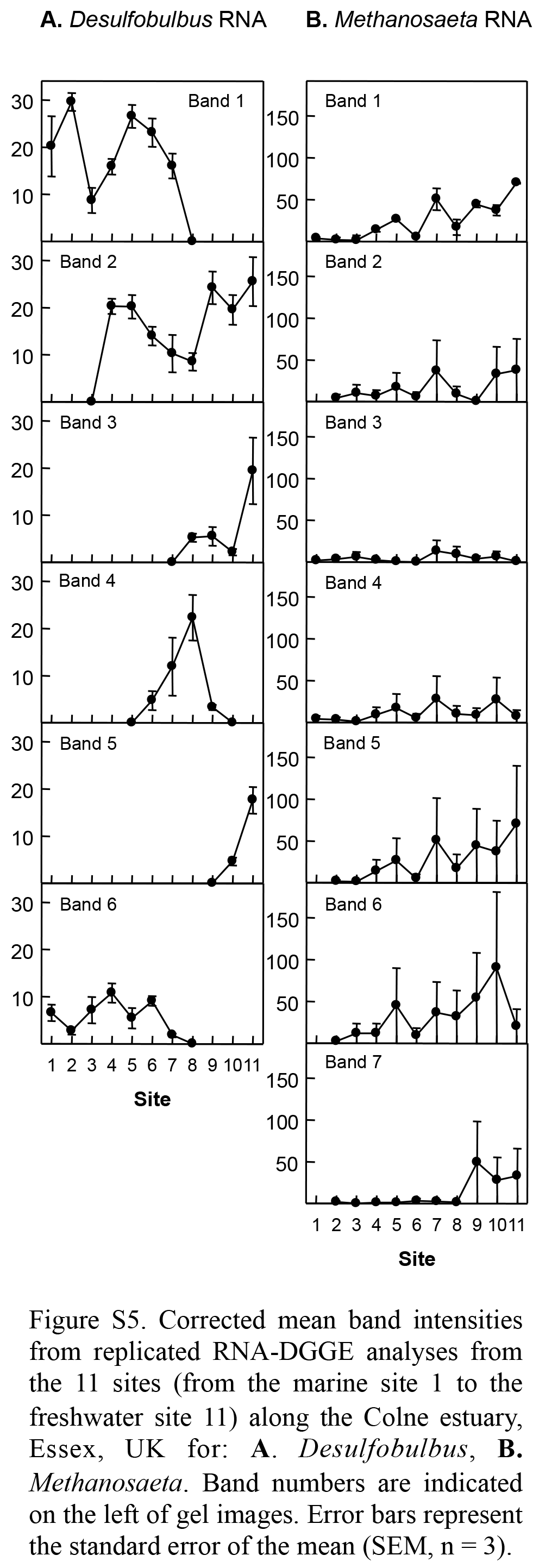

Supplement: Figure S5 — Corrected mean band intensities from replicated RNA-DGGE analyses from the 11 sites (from the marine site 1 to the freshwater site 11) along the Colne estuary, Essex, UK for: A. Desulfobulbus , B. Methanosaeta . Band numbers are indicated on the left of gel images. Error bars represent the standard error of the mean (SEM, n = 3). (TIF) [file pone.0085105.s005.tif]

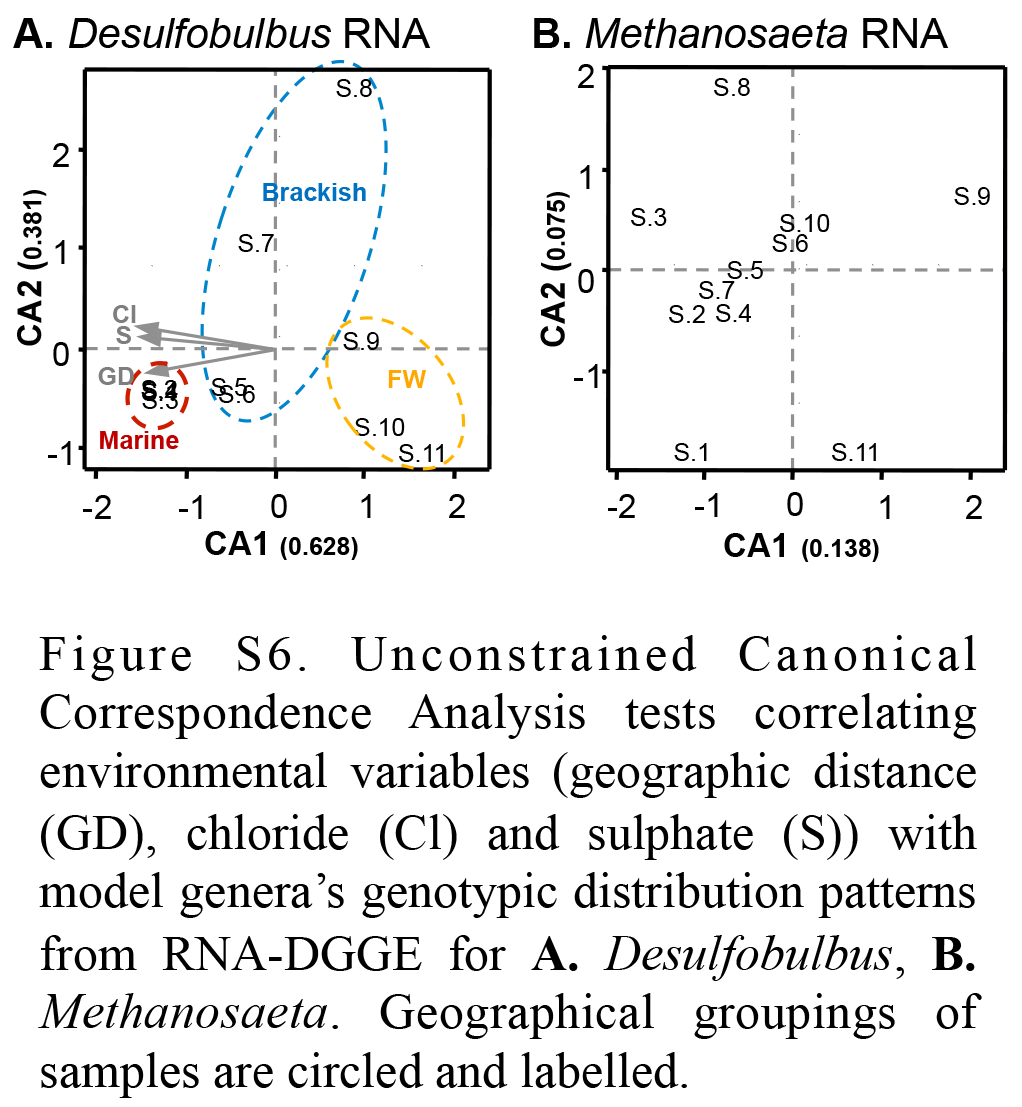

Supplement: Figure S6 — Unconstrained Canonical Correspondence Analysis tests correlating environmental variables (geographic distance (GD), chloride (Cl) and sulphate (S)) with model genera's genotypic distribution patterns from RNA-DGGE for A. Desulfobulbus , B. Methanosaeta . Geographical groupings of samples are circled and labelled. (TIF) [file pone.0085105.s006.tif]
