# Supplementary material for: Metabolic Flexibility as a Major Predictor of Spatial Distribution in Microbial Communities
Source: PLoS One. 2014 Jan 21;9(1):e85105. doi: 10.1371/journal.pone.0085105 (PMC3897421; doi:10.1371/journal.pone.0085105)
Supplement: File S1 — Contains Table S1–S3 and detailed description of Materials and Methods used and basic results with associated references. Table S1: List of PCR primers and annealing temperatures used in this study. *TD - These PCRs were Touchdown PCRs. Initial annealing temperature is +10°C and reduces by 1°C per cycle for the first 10 cycles of the PCR. Table S2. Mantel tests correlating environmental variables (geographic distance (D), chloride (C) and sulphate (S)) with model genera's DNA-DGGE based genotypic distribution patterns (based on Band intensities, Pearson and Jaccard analyses). Table S3. Mantel tests correlating environmental variables (geographic distance (GD), chloride (C) and sulphate (S)) with model genera's RNA-DGGE based genotypic distribution patterns (based on Band intensities, Pearson and Jaccard analyses). (DOCX) [file pone.0085105.s007.docx]

**Supplementary Information S1**

**Material and Methods**

Site description and sampling

The Colne estuary is a small, muddy, macrotidal estuary on east coast of the UK, entering the North Sea at Brightlingsea. The estuary catchment is 500 km^2^, of which the River Colne drains 300 km^2^, much of which is rich arable land. Triplicates sediment cores (10 cm diameter) were taken at low tide from 11 sites (numbered from 1 (marine) to 11 (freshwater)) in September 2007 (see Fig. S1; [[1](#_ENREF_1)]). Sediment samples, from the sediment surface to 5 cm deep (0-5 cm depth horizon), were taken from the sediment cores using a 5 ml syringe with the luer cut-off (4 mini-cores from each larger sediment core were amalgamated into one sample) were centrifuged at 4000*g* for 3 minutes to collect pore water. The sediment was immediately washed in 120mM sodium phosphate, pH 8.0, to remove extracellular DNA [[2](#_ENREF_2)], then centrifuged at 4000*g* for 3 minutes and the supernatant discarded. The washed sediment were immediately divided into three roughly equal subsamples, placed into foil packets and stored frozen at -20°C in order to avoid RNA degradation.

Porewater sulphate, chloride and nitrate concentrations in sediment samples taken in 2004 were measured by ion chromatography, using a Dionex DX-500 HPIC fitted with an AS14 separator column (Dionex Corp., California, USA) (see Fig. S2; [[1](#_ENREF_1)]). A coherent decreasing gradient was shown for chloride and sulphate. Acetate concentrations in sites 1 and 11 have been measured previously [[3](#_ENREF_3)] and range from 19-26 µM at Site 1 and 19-100 µM at Site 11.

Nucleic acids extraction from sediments

DNA and RNA were extracted separately directly from sediment samples using the hydroxyapatite spin-column method [[4](#_ENREF_4),[5](#_ENREF_5)]. Some DNA and RNA extracts were cleaned up by precipitation in 1 volume of 30% w/v PEG6000 and 0.1 volume of 5M NaCl to purify the nucleic acids further [[5](#_ENREF_5),[6](#_ENREF_6)]. DNA extraction yield was determined by spectrophotometry (Nanodrop, Thermo Scientific, UK) and ethidium bromide-stained agarose gel electrophoresis was used to visually check DNA and RNA quality.

PCR amplification of 16S rRNA gene sequences from the model genera

The primers used in this study and their relative annealing temperature and supporting references are listed in Table S1. All PCR amplifications were performed using a Mastercycler^®^ or MastercyclerPro^®^ (Eppendorf, UK). Wide specificity primers for Archaea (1Af/1404r), Euryarchaea (1Af/1100r) and Bacteria (8f/1541r) were used in a nested or semi-nested combination with genera specific primers (Dbb121f/Dbb1237r for *Desulfobulbus*, DSB127/DSB1273 for *Desulfobacter* [[7](#_ENREF_7)] and 1Af/ Mst 746r for *Methanosaeta* [[8](#_ENREF_8),[9](#_ENREF_9)]) as well as DGGE primers for Archaea or Bacteria (Table S1). Generally, the PCR programs used were a 2 min 96°C hot start, 10 cycles of denaturation (96°C for 1 min), annealing (annealing temperature for 1min) and elongation (72°C for 2.5 min), then 20 cycles of denaturation (96°C for 30s), annealing (annealing temperature for 30s) and elongation (72°C for 2 min) and finally 7 min elongation at 72°C. For the general Archaea (1Af/1404R; Table S1) and the DGGE primer pairs, a touchdown PCR was used in the first 10 cycles, with initial annealing temperature 10°C higher than the annealing temperature given in Table 1 which then decreases by 1°C per PCR cycle until it reached the stated annealing temperature. PCR amplifications were performed with GoFlexiTaq (Promega, UK) as per manufacturer recommendations, with 0.2 pM of each primer. Templates were added at a 1/50 ratio in the PCR reactions.

Design of *Methanosarcina* specific primers

A new set of primers specific for *Methanosarcina* 16S rRNA gene (Msc214f and Msc613r; Table 1) was designed using the web-based primer design software Primer3 (http://frodo.wi.mit.edu/primer3/), and assessed for specificity and sensitivity with ThermoPhyl (a Perl script programme that assesses multiple potential PCR assays, http://go.warwick.ac.uk/thermophyl [[10](#_ENREF_10)]). Specificity was checked empirically by amplification of *Methanosarcina mazei* (DSM 2053) and *Methanosarcina acetivorans* (DSM 2834) pure culture DNA (extracted with a Blood and Tissue DNA kit, Invitrogen) and on several other methanogen, archaeal and bacterial negative controls.

Nested and semi-nested PCR approaches

In order to amplify specific *Methanosaeta* PCR products from all sediment samples. Using the primers listed in Table 1, an initial general archaeal PCR (1Af/1404r) was performed, then a euryarchaeal PCR (1Af/1100r) as described by Munson *et al.* [[11](#_ENREF_11)], then a *Methanosaeta* specific PCR (1Af/Mst746r), and finally a fragment for Denaturing Gradient Gel Electrophoresis (DGGE) was amplified [[12](#_ENREF_12)]. It was possible to obtain *Methanosaeta*-specific PCR products from freshwater sediments with only 2 rounds of PCR but 4-steps were required to amplify products from the marine sediments therefore this 4-step protocol was used for all samples. Such a protracted PCR amplification may be prone to biases but similarity in distribution patterns between this 4-step DNA-DGGE approach and the 2-step RNA-DGGE (see below) suggest that this amplification was not subject to substantial bias. A similar approach, as used by Hawkins & Purdy [[1](#_ENREF_1)], was used for both *Desulfobulbus* and *Desulfobacter*. Here, the bacterial (8f/1541r) then either the *Desulfobulbus* (Dbb121f/Dbb1237r) or *Desulfobacter* (DSB127f/DSB1273r) specific primers and finally the Bacteria DGGE primers (PRBA340f-GC/PRUN518r) were sequentially amplified. The efficacy of this approach has been validated by Dar *et al.* [[13](#_ENREF_13)]

Reverse-transcriptase PCR

The active community of *Desulfobulbus* and *Methanosaeta* were analysed using reverse-transcript PCR (RT-PCR). RNA extracts, for use in RT-PCR experiments, were incubated using DNaseQ (Invitrogen, UK) as per manufacturer instructions to remove any remnant DNA. Reverse transcription was performed using Superscript II (Invitrogen, UK) as per manufacturer recommendations, using the reverse primer targeting the genus of interest (Dbb1237r for *Desulfobulbus* and Mst746r for *Methanosaeta*). The resultant cDNAs were then used as templates for genus specific PCR following protocols described above, and used as templates for DGGE PCR. DNAse-treated RNA from each individual sample was used as a negative control to ensure that no amplifiable DNA was present in the RNA.

DGGE analyses of amplified 16S rRNA gene sequence from the model genera

The final PCR products from each genus were analysed on 8% (w/v) acrylamide/bis-acrylamide (37.5:1; 40% w/v) gels with a 20-60% denaturant gradient according to the method described by Schafer & Muyzer [[14](#_ENREF_14)]. The HyperLadder I (Bioline, UK) was used as reference in all gels. DGGE were run for 16 hours at 75 V and 60°C, stained with ethidium bromide and then visualised by UV transillumination.

Roughly half of the bands from each gel were excised and re-amplified using the relevant DGGE primers for sequence analysis [[14](#_ENREF_14)] to assess the specificity of this DGGE approach for each genus.

Gel images were analysed in the software package GelComparII (Applied Maths) using Hyperladder I (Bioline, UK) as a reference to compare band migration across samples and gels. Both relative band intensities of selected bands and a Pearson analysis of the whole profile of each lane were determined. Cluster analyses in GelComparII were performed with both the Jaccard correlation analysis (based on band presence/absence similarity matrices) and the Pearson correlation analysis (based on the intensity curve along each sample lane).

Statistical analyses

Dissimilarity matrices were obtained from Pearson and Jaccard analyses in GelComparII, and calculated from band intensities values in R (The R Project for statistical computing, http://www.r-project.org/index.html). Environmental variables (geographic distance, chloride and sulphate porewater concentrations) were also converted into dissimilarity matrixes. In addition, mantel and partial mantel tests [[15](#_ENREF_15)], which test the correlation between two matrixes, were performed using these different genotype and environmental similarity matrices.

Canonical Correspondence Analyses (CCA) are widely used to assess the effect of an environmental gradient on the distribution of organisms, that allow multiple regression with multiple environmental variables [[16](#_ENREF_16)]. CCAs and fitting of environmental vectors were performed in R using the vegan library [[17](#_ENREF_17)].

Results

Nucleic acids extraction and PCR amplification

DNA and RNA were successfully extracted from all 33 samples. *Methanosarcina*-targeted primer pair specificity was confirmed as amplification was only obtained from pure cultures of *Methanosarcina* and not from other related methanogens or various bacterial and archaeal controls (data not shown). Along with the *Methanosarcina* analysis, PCR amplicons of the correct sizes were obtained for *Desulfobulbus*, *Desulfobacter* and *Methanosaeta* specific primer pairs after their respective nested PCRs and were successfully amplified for DGGE analyses.

DGGE

Mean corrected values for band intensity were relatively constant with some variability between replicates. Sequences from excised DGGE bands were all closely related to previously sequenced *Methanosaeta*, *Methanosarcina*, *Desulfobulbus* or *Desulfobacter* isolates and clones in each case.

The accuracy of molecular fingerprinting methods have been questioned, especially when studying whole communities [[18](#_ENREF_18)]. In this case, the analyses were limited to single genera and thus should have produced a good representation of the actual distributions. We have shown for *Desulfobulbus* [[1](#_ENREF_1),[19](#_ENREF_19)] and for *Methanosaeta* [[9](#_ENREF_9)] that DGGE profiles actually produce comparable results to more extensive molecular methods (clone libraries analyses, group specific qPCR assays [[19](#_ENREF_19)] and pyrosequence analysis [[20](#_ENREF_20),[21](#_ENREF_21)]).

References

1. Hawkins RJ and Purdy KJ (2007) Genotypic distribution of an indigenous model microorganism along an estuarine gradient. FEMS Microbiol Ecol 62: 187-194.

2. Tsai YL and Olson BH (1991) Rapid method for direct extraction of DNA from soil and sediments. Appl Environ Microbiol 57: 1070-1074.

3. Silva S (2004) Activity and diversity of sulphate-reducing bacteria and methanogenic archaea in contrasting sediments from the River Colne estuary. Colchester, UK: University of Essex.

4. Purdy KJ, Embley TM, Takii S and Nedwell DB (1996) Rapid extraction of DNA and rRNA from sediments by a novel hydroxyapatite spin-column method. Appl Environ Microbiol 62: 3905-3907.

5. Purdy KJ (2005) Nucleic acid recovery from complex environmental samples. Meth Enzymol 397: 271-292.

6. Selenska S and Klingmüller W (1991) DNA recovery and direct detection of *Tn5* sequences from soil. Lett Appl Microbiol 13: 21-24.

7. Daly K, Sharp RJ and McCarthy AJ (2000) Development of oligonucleotide probes and PCR primers for detecting phylogenetic subgroups of sulfate-reducing bacteria. Microbiology 146: 1693-1705.

8. Carbonero F, Oakley BB and Purdy KJ (2010) Improving the isolation of anaerobes on solid media: The example of the fastidious *Methanosaeta*. J Microbiol Meth 80: 203-205.

9. Carbonero F, Oakley BB and Purdy KJ (2012) Genotypic distribution of a specialist model microorganism, *Methanosaeta*, along an estuarine gradient: Does metabolic restriction limit niche differentiation potential? Microb Ecol 63: 856-864.

10. Oakley BB, Dowd SE and Purdy KJ (2011) ThermoPhyl: A software tool for designing thermodynamically and phylogenetically optimized quantitative-PCR assays. FEMS Microbiol Ecol 77: 17-27.

11. Munson MA, Nedwell DB and Embley TM (1997) Phylogenetic diversity of Archaea in sediment samples from a coastal salt marsh. Appl Environ Microbiol 63: 4729-4733.

12. Øvreås L, Forney L, Daae FL and Torsvik V (1997) Distribution of bacterioplankton in meromictic Lake Saelenvannet, as determined by denaturing gradient gel electrophoresis of PCR-amplified gene fragments coding for 16S rRNA. Appl Environ Microbiol 63: 3367-3373.

13. Dar SA, Kuenen JG and Muyzer G (2005) Nested PCR-Denaturing Gradient Gel Electrophoresis approach to determine the diversity of sulfate-reducing bacteria in complex microbial communities. Appl Environ Microbiol 71: 2325-2330.

14. Schäfer H and Muyzer G (2001) Denaturing Gradient Gel Electrophoresis in marine microbial ecology. Meth Microbiol 30: 425-468.

15. Mantel N and Fleiss JL (1980) Minimum expected cell-size requirements for the Mantel-Haenszel one-degree-of-freedom chi-square test and a related rapid procedure. Amer J Epidemiol 112: 129-134.

16. Peres-Neto PR, Legendre P, Dray S and Borcard D (2006) Variation partitioning of species data matrices: Estimation and comparison of fractions. Ecology 87: 2614-2625.

17. Dixon P (2003) VEGAN, a package of R functions for community ecology. J Veget Sci 14: 927-930.

18. Bent SJ and Forney LJ (2008) The tragedy of the uncommon: understanding limitations in the analysis of microbial diversity. ISME J 2: 689-695.

19. Oakley BB, Carbonero F, van der Gast CJ, Hawkins RJ and Purdy KJ (2010) Evolutionary divergence and biogeography of sympatric niche-differentiated bacterial populations. ISME J 4: 488-497.

20. Oakley BB, Carbonero F, Dowd SE, Hawkins RH and Purdy KJ (*In press*) Contrasting patterns of niche partitioning between two anaerobic terminal oxidizers of organic matter. ISME J.

21. Purdy KJ, Hurd PJ, Moya-Laraño J, Trimmer M, Oakley BB, et al. (2011) Systems biology for ecology: From molecules to ecosystems. Adv Ecolog Res 43: 88-149.

22. Embley TM, Finlay BJ, Thomas RH and Dyal PL (1992) The use of rRNA sequences and fluorescent probes to investigate the phylogenetic positions of the anaerobic ciliate *Metopus palaeformis* and its archaeobacterial endosymbiont. J Gen Microbiol 138: 1479-1487.

23. Embley TM (1991) The linear PCR reaction - A simple and robust method for sequencing amplified rRNA genes. Lett Appl Microbiol 13: 171-174.

Table S1: List of primers and annealing temperatures used in this study. ^*^TD - These PCRs were Touchdown PCRs. Initial annealing temperature is +10°C and reduces by 1°C per cycle for the first 10 cycles of the PCR.

| Primer pairs | Sequence 5’-3’ | Annealing temp (°C) | Reference |
| --- | --- | --- | --- |
| Archaea  1Af  1404r | TCYGKTTGATCCYGSCRGAG  CGGTGTGTGCAAGGRGC | 53 (TD^*^) | [[22](#_ENREF_22)]  [[11](#_ENREF_11)] |
| Euryarchaea  1Af  1100r | TCYGKTTGATCCYGSCRGAG  TGGGTCTCGCTCGTTG | 50 | [[22](#_ENREF_22)] |
| *Methanosarcina*  Msc214f  Msc613r | TCTGCGGCCTATCAGGTAGT  GGAACCGGGAGAGGTAAGAG | 56 | This study |
| *Methanosaeta*  1Af  Mst746r | TCYGKTTGATCCYGSCRGAG  GTCCCTTGCCGTCAGGTC | 67 | [[22](#_ENREF_22)]  [[8](#_ENREF_8)] |
| DGGE Archaea  PARCH340f-GC  PARCH519r | CGCCCGCCGCGCGCGGCGGGCGGGGCGGGGGCACGGGGGGCCCTACGGGGYGCASCAG  TTACCGCGGCKGCTG | 53.5 (TD^*^) | [[12](#_ENREF_12)] |
| Bacteria  8f  1541r | AGAGTTTGATCCTGGCTCAG  AAGGAGGTGATCCAGCCGCA | 50 | [[23](#_ENREF_23)] |
| *Desulfobulbus*  DBB121f  DBB1237r | CGCGTAGATAACCTGTCYTCATG  GTAGKACGTGTGTAGCCCTGGTC | 59 | [[7](#_ENREF_7)]  [[1](#_ENREF_1)] |
| *Desulfobacter*  DSB127f  DSB1273r | GATAATCTGCCTTCAAGCCTGG  CYYYYYOCRRAGTCGSTGCCCT | 60 | [[7](#_ENREF_7)] |
| DGGE Bacteria  PRBA340f-GC  PRUN518r | CGCCCGCCGCGCGCGGCGGGCGGGGCGGGGGCACGGGGGGACTCCTACGGGAGGCAGCAG  ATTACCGCGGCTGCTGG | 60 (TD^*^) | [[12](#_ENREF_12)] |

Table S2. Mantel tests correlating environmental variables (geographic distance (D), chloride (C) and sulphate (S)) with model genera’s DNA-DGGE based genotypic distribution patterns (based on Band intensities, Pearson and Jaccard analyses).

| **A.** *Desulfobulbus* | | | Band intensity | Jaccard | Pearson |
| --- | --- | --- | --- | --- | --- |
| Mantel | Distance | | **0.001** | **0.001** | **0.001** |
|  | Chloride | | **0.001** | **0.001** | **0.001** |
|  | Sulphate | | **0.002** | **0.002** | **0.002** |
| Partial Mantel | Distance / Chloride | | **0.006** | **0.045** | 0.206 |
|  | Distance / Sulphate | | **0.004** | **0.010** | **0.032** |
|  | Chloride / Distance | | 0.418 | 0.051 | **0.028** |
|  | Chloride / Sulphate | | 0.410 | 0.070 | 0.084 |
|  | Sulphate / Distance | | **0.048** | 0.404 | 0.220 |
|  | Sulphate / Chloride | | **0.021** | **0.036** | **0.011** |
| **B.** *Methanosarcina* | | | Band intensity | Jaccard | Pearson |
| Mantel | Distance | | **0.002** | **0.002** | **0.001** |
|  | Chloride | | **0.008** | **0.009** | **0.003** |
|  | Sulphate | | **0.002** | **0.007** | **0.001** |
| Partial Mantel | Distance / Chloride | | **0.015** | **0.004** | **0.025** |
|  | Distance / Sulphate | | **0.031** | **0.009** | 0.086 |
|  | Chloride / Distance | | 0.919 | 0.976 | 0.635 |
|  | Chloride / Sulphate | | 0.858 | 0.828 | 0.821 |
|  | Sulphate / Distance | | 0.273 | 0.613 | 0.143 |
|  | Sulphate / Chloride | | **0.010** | **0.050** | **0.012** |
| **C.** *Methanosaeta* | | | Band intensity | Jaccard | Pearson |
| Mantel | Distance | | 0.119 | 0.139 | 0.144 |
|  | Chloride | | 0.263 | 0.183 | 0.316 |
|  | Sulphate | | 0.317 | 0.159 | 0.291 |
| Partial Mantel | Distance / Chloride | | **0.011** | 0.148 | **0.001** |
|  | Distance / Sulphate | | **0.020** | 0.327 | 0.087 |
|  | Chloride / Distance | | 0.913 | 0.647 | 0.935 |
|  | Chloride / Sulphate | | 0.285 | 0.803 | 0.325 |
|  | Sulphate / Distance | | 0.902 | 0.320 | 0.794 |
|  | Sulphate / Chloride | | 0.686 | 0.100 | 0.488 |
| **D.** *Desulfobacter* | | | Band intensity | Jaccard | Pearson |
| Mantel | | Distance | 0.396 | 0.139 | 0.144 |
|  | | Chloride | 0.512 | 0.183 | 0.316 |
|  | | Sulphate | 0.437 | 0.159 | 0.291 |
| Partial Mantel | | Distance / Chloride | 0.151 | **0.008** | 0.327 |
|  |  | Distance / Sulphate | 0.335 | **0.045** | 0.732 |
|  | | Chloride / Distance | 0.850 | 0.900 | 0.702 |
|  | | Chloride / Sulphate | 0.812 | 0.512 | 0.995 |
|  | | Sulphate / Distance | 0.538 | 0.680 | 0.219 |
|  | | Sulphate / Chloride | 0.230 | 0.312 | **0.009** |

Table S3. Mantel tests correlating environmental variables (geographic distance (DISTANCE), chloride (C) and sulphate (S)) with model genera’s RNA-DGGE based genotypic distribution patterns (based on Band intensities, Pearson and Jaccard analyses).

| **A.** *Desulfobulbus* | | Band intensity | Jaccard | Pearson |
| --- | --- | --- | --- | --- |
| Mantel | Distance | **0.001** | **0.001** | 0.158 |
|  | Chloride | **0.001** | **0.001** | 0.227 |
|  | Sulphate | **0.001** | **0.004** | 0.229 |
| Partial Mantel | Distance / Chloride | **0.006** | **0.001** | 0.095 |
|  | Distance / Sulphate | **0.005** | **0.001** | 0.210 |
|  | Chloride / Distance | 0.793 | 0.996 | 0.654 |
|  | Chloride / Sulphate | 0.478 | 0.066 | 0.647 |
|  | Sulphate / Distance | 0.351 | 0.933 | 0.373 |
|  | Sulphate / Chloride | 0.082 | 0.533 | 0.177 |
| **B.** *Methanosaeta* | | Band intensity | Jaccard | Pearson |
| Mantel | Distance | **0.007** | **0.001** | **0.024** |
|  | Chloride | **0.008** | **0.001** | **0.012** |
|  | Sulphate | **0.001** | **0.003** | **0.013** |
| Partial Mantel | Distance / Chloride | 0.062 | 0.447 | 0.836 |
|  | Distance / Sulphate | 0.134 | 0.253 | 0.494 |
|  | Chloride / Distance | 0.705 | **0.021** | **0.030** |
|  | Chloride / Sulphate | 0.927 | 0.325 | 0.284 |
|  | Sulphate / Distance | 0.107 | **0.022** | **0.028** |
|  | Sulphate / Chloride | **0.006** | 0.089 | 0.194 |
